# Morphological and phylogenetic analyses reveal three new species of *Neosarcodon* (*Thelephorales*, *Basidiomycota*) from China

**DOI:** 10.3897/mycokeys.141.202277

**Published:** 2026-09-30

**Authors:** Xin-Yue Yang, Li-Qing Deng, Ying-Ming Zhang, Wang-Qiu Deng, Hai-Mei Yue, Chao-Qun Wang, Ming Zhang

**Affiliations:** 1 State Key Laboratory of Applied Microbiology Southern China, Guangdong Provincial Key Laboratory of Microbial Culture, Collection and Application, Institute of Microbiology, Guangdong Academy of Sciences, Guangzhou 510070, China State Key Laboratory of Applied Microbiology Southern China, Guangdong Provincial Key Laboratory of Microbial Culture, Collection and Application, Institute of Microbiology, Guangdong Academy of Sciences Guangzhou China; 2 College of Plant Science, Xizang Agricultural and Animal Husbandry University, Linzhi 860000, China College of Plant Science, Xizang Agricultural and Animal Husbandry University Linzhi China; 3 College of Horticulture, Ludong University, Yantai 264000, China College of Horticulture, Ludong University Yantai China; 4 Guangdong Chebaling National Nature Reserve Administration, Shaoguan 512526, China Guangdong Chebaling National Nature Reserve Administration Shaoguan China; 5 College of Biology and Food Engineering, Guangdong University of Education, Guangzhou 510303, China College of Biology and Food Engineering, Guangdong University of Education Guangzhou China; 6 Guangdong Nanling Forest Ecosystem National Observation and Research Station, Shaoguan 510735, China Guangdong Nanling Forest Ecosystem National Observation and Research Station Shaoguan China

**Keywords:** New taxon, phylogeny, *
Sarcodonaceae
*, taxonomy

## Abstract

The species diversity and phylogenetic relationships of the genus *Neosarcodon* in subtropical regions of southern China remain insufficiently explored. In this study, three new *Neosarcodon* species are illustrated and described from the subtropical regions of southern China, based on both morphological evidence and multigene phylogenetic analyses by the internal transcribed spacer regions (nrITS), the large subunit of nuclear ribosomal RNA gene (nrLSU) and the second largest subunit of RNA polymerase II (*rpb2*). *Neosarcodon chebalingensis* is characterised by its orange-grey to brownish-grey basidiomata with a glabrous to subtomentose pileus, orange-grey spines changing to bluish-black when bruised, subglobose basidospores, measuring 7.5–10.5 × (7)7.5–9.5(10) μm; *Neosarcodon nanlingensis* is characterised by its grey to greyish-brown basidiomata with glabrous pileus, dark-blond to oak-brown spines, changing to dark-grey when bruised, subglobose basidospores measuring 8–10(11) × (7.5)8–9.5(10) μm; *Neosarcodon velutinus* is characterised by its violet-grey to dark-violet basidiomata with a velvety pileus, golden-grey to brownish-grey spines changing to dull-violet to greyish-violet when bruised, subglobose to broadly ellipsoid basidospores measuring 6.5–8.5(9) × 5.5–8 μm. Phylogenetic analysis well supported the three species as new members of *Neosarcodon*. A key to the Chinese *Neosarcodon* species was also provided in the present study. This study enriches the documented species diversity of the genus and expands our understanding of morphological variation within *Neosarcodon*.

## Introduction

*Neosarcodon* Xiao L. He, Di Wang & W.H. Peng, typed by *N. pakaraimensis* (A. Grupe & T.W. Henkel) Xiao L. He, Di Wang & W.H. Peng, was recently established in order to accommodate the “*Neosarcodon* clade” in Grupe et al. (2015, 2016) and Larsson et al. (2019) (Wang et al. 2024). Species of the genus are mainly characterised by stipitate basidiomata; broadly conic to plano-convex or nearly applanate pileus with glabrous to fibrillose surface; hollow to solid stipe, usually concolorous with the pileus; adnate to nearly free spines, white or pallid at first, becoming brownish-grey or blackish with age; bitter tasting context, whitish to pale greyish at first, unchanging or changing to reddish to blackish when exposed; subglobose, brownish and tuberculate basidiospores; thin-walled hyphae with abundant clamp connections; and the absence of hymenial cystidia (Grupe et al. 2015, 2016; Larsson et al. 2019; Wang et al. 2024). Before the establishment of *Neosarcodon*, species with those characteristics were mainly classified in *Sarcodon* and treated as the “*Neosarcodon* clade” (Grupe et al. 2015, 2016; Larsson et al. 2019). Recently, based on a multilocus (nrITS-nrLSU-*rpb2*) phylogenetic study, the “*Neosarcodon* clade” was formally upgraded to genus level and demonstrated that species of *Neosarcodon* mainly differs from *Sarcodon* by having the adnate hymenophore, indistinct odour and the absence of hymenial cystidia (Wang et al. 2024). Subsequently, a systematic revision of *Thelephorales* proved that *Neosarcodon* belongs to the newly-established family *Sarcodonaceae* within *Thelephorales* (Song et al. 2025).

Ecologically, most known *Neosarcodon* species are ectomycorrhizal fungi and can form symbiotic relationships with numerous trees, such as *Caesalpinioideae*, *Dipterocarpaceae*, *Fagaceae* and *Papilionoideae* (Grupe et al. 2015, 2016). However, *N. carbonarius* (Maas Geest.) J.A. Cooper & T.W. May, originally reported from Stewart Island and frequently occurring in pure podocarp stands, has been hypothesised to be non-ectomycorrhizal. Therefore, its trophic status remains uncertain and requires further investigation (Douch et al. 2024).

Up to now, 13 species of *Neosarcodon* have been reported worldwide. They are mainly distributed in the Neotropics (Douch et al. 2024; Wang et al. 2024). Amongst them, 10 are found in the New Tropical Region, but there are still some exceptions, three species having been discovered from the subtropical. *Neosarcodon atroviolaceus* L.J. Zhou, Y.Q. Zhu & H.S. Yuan, *N. bambusicola* L.J. Zhou, Y.Q. Zhu & H.S. Yuan and *N. melanocarpus* B.K. Cui, Y.F. Sun & C.G. Song were recently reported from subtropical regions of China (Song et al. 2025; Zhu et al. 2026).

During a survey of macrofungi diversity in Guangdong Province, several distinct stipitate hydnoid samples were collected. Further study proved that they represent three species of *Neosarcodon*, new to science, which are formally introduced in the present study.

## Materials and methods

### Sample collection and morphological study

In the field, fresh specimens were photographed and detailed morphological characteristics and habitat information were recorded. The specimens were dried using an electric dryer at about 40 °C and then deposited in the Fungarium of Institute of Microbiology, Guangdong Academy of Sciences, Guangzhou, China (GDGM). Macro-morphological characteristics of samples were described, based on field notes and photographs, colour codes and colour descriptions of the samples were derived from Kornerup and Wanscher (1978). Microscopic observations were carried out on dried tissue sections rehydrated in 5% potassium hydroxide (KOH) and stained with 1% aqueous Congo red or Melzer’s reagent under a light microscope. Microphotographs and measurements were obtained using a light microscope (ECLIPSE Ni-U, Nikon Corporation, Japan) at magnifications ranging from 100× to 1000×.

For basidiospore descriptions, a notation (a)b–c(d) describes the measured basidiospore dimension (including ornamentation), where the range “b–c” represents 90% or more of the measured values, “a” and “d” are the extreme values; Lm and Wm indicate the average length and width (± standard deviation) of the measured basidiospores, respectively; Q value refer to the length/width ratio of an individual basidiospore and Qm refers to the average Q value of all measured basidiospores ± standard deviation.

### DNA extraction, PCR amplification and sequencing

Genomic DNA solutions were obtained from silica gel dried tissue utilising a HiPure Fungal DNA Kit (Magen Biotechnology Co., Ltd., Guangzhou, China). The nrITS, nrLSU and *rpb2* were amplified using the primers ITS1/ITS4, LR0R/LR7 and bRPB2-6F/bRPB2-7.1R, respectively (Vilgalys and Hester 1990; White et al. 1990). Polymerase chain reaction (PCR) was performed following the protocol of Zhang et al. (2025). PCR amplification products were verified by agarose gel electrophoresis and sent to Tianyi Huiyuan Gene Technology Co., LTD. (Guangzhou, China) for sequencing using the same primers as the PCR reaction, respectively.

### Phylogenetic analyses

The raw DNA sequences were assembled and trimmed using Geneious Prime 2025.0.2 (www.geneious.com), then compared with sequences in the NCBI database using the Nucleotide BLASTn tool to ensure data accuracy. Newly-generated sequences were submitted to GenBank and assigned accession numbers (Table 1). Representative sequences were retrieved from GenBank following Douch et al. (2024), Wang et al. (2024), Song et al. (2025) and Zhu et al. (2026), with *Amaurodon aquicoeruleus* Agerer selected as the outgroup (Douch et al. 2024; Wang et al. 2024). The nrITS, nrLSU and *rpb2* datasets were aligned using the auto strategy in MAFFT v.7.505 (Katoh and Standley 2013) and manually modified in MEGA v.6 (Tamura et al. 2013). The three aligned datasets were concatenated into a single dataset using the “Concatenate Sequence” option in PhyloSuite v.2 (Zhao et al. 2025). The best-fit models for Bayesian Inference (BI) and Maximum Likelihood (ML) analyses were determined using ModelFinder v.2.2.0 (Kalyaanamoorthy et al. 2017). ML analysis was conducted in IQ-TREE (Minh et al. 2013; Nguyen et al. 2015) as implemented in PhyloSuite v.2 (Zhao et al. 2025), with 5000 ultrafast bootstrap replicates to assess nodal support and a minimum support threshold of 0.90. Bayesian Inference (BI) was performed using MrBayes v.3.2.7a (Ronquist et al. 2012), excluding the first 25% of sampled data as burn-in. The phylogenetic trees were visualised using FigTree v.1.4.4 (Rambaut 2018) and edited in Adobe Illustrator 2023. In this study, the tree topology of ML was used as a representative and the bootstrap values marked on the clade were significantly credible: the Maximum Likelihood Bootstrap Support (MLBS) not less than 75% and the Bayesian Posterior Probability (BPP) not less than 0.95.

**Table 1.** Taxa, voucher numbers, localities, GenBank accession numbers and references of samples used in phylogenetic analyses are provided. Holotypes are indicated as HT and epitypes as ET. Newly-generated sequences are presented in bold.

**Taxon**	**Voucher number**	**Locality**	**GenBank accession numbers**			**Reference**
			**nrITS**	**nrLSU**	***rpb2***	
*Amaurodon aquicoeruleus*	UK452	Australia	AM490944	AM490944	**–**	Miettinen and Kõljalg (2007)
*Hydnellum bomiense*	Yuan13759	China	MW579941	MW579887	OK254206	Mu et al. (2021)
*H. brunneorubrum*	Yuan14668	China	MW579945	MW579890	OK254218	Mu et al. (2021)
*H. edulium*	SAAS 2727 (HT)	China	OK636093	OP407675	OP434358	Wang et al. (2024)
*H. edulium*	SAAS 2870 (ET)	China	OK636094	OP407676	OP434359	Wang et al. (2024)
*H. edulium*	SAAS 2920 (ET)	China	OP437910	OP407677	OP434360	Wang et al. (2024)
*H. illudens*	SAAS 3830	China	OP437912	OP407678	OP434361	Wang et al. (2024)
*H. illudens*	SAAS 3838	China	OP437913	OP407679	OP434362	Wang et al. (2024)
*H. illudens*	SAAS 3844	China	OP437914	OP407680	OP434363	Wang et al. (2024)
*H. subalpinum*	SAAS 2778 (HT)	China	OP437919	OP407685	OP434369	Wang et al. (2024)
*H. subalpinum*	SAAS 2884 (ET)	China	OP437920	OP407686	OP434370	Wang et al. (2024)
*H. subalpinum*	SAAS 2923 (ET)	China	OP437921	OP407687	OP434371	Wang et al. (2024)
*H. subalpinum*	SAAS 2961	China	OP437922	OP407688	OP434372	Wang et al. (2024)
*H. versipelle*	SAAS 2841	China	OP437923	OP407689	**–**	Wang et al. (2024)
*H. versipelle*	SAAS 2905	China	OP437924	OP407690	**–**	Wang et al. (2024)
***Neosarcodon atroviolaceus***	**GDGM46168**	**China (Guangdong)**	**PZ326305**	**PZ317568**	**–**	**This study**
***N. atroviolaceus***	**GDGM91284**	**China (Guangdong)**	**PZ326308**	**PZ317570**	**PZ339035**	**This study**
***N. atroviolaceus***	**GDGM94993**	**China (Guangdong)**	**PZ326309**	**PZ317571**	**–**	**This study**
***N. atroviolaceus***	**GDGM98720**	**China (Guang dong)**	**PZ326312**	**PZ317574**	**PZ339038**	**This study**
***N. atroviolaceus***	**GDGM99604**	**China (Guangdong)**	**PZ326315**	**PZ317577**	**PZ339041**	**This study**
*N. atroviolaceus*	Yuan19330 (HT)	China	PV221948	PV257886	**–**	Zhu et al. (2026)
*N. atroviridis*	REB-109	USA	KC571769	**–**	KF007966	Direct Submission
*N. bairdii*	AV990 (HT)	Colombia	KR698938	**–**	**–**	Grupe et al. (2016)
*N. bambusicola*	Yuan19491 (HT)	China	PV221949	PV257884	**–**	Zhu et al. (2026)
*N. carbonarius*	PDD:113342	New Zealand	MW683597	MW683924	**–**	Douch et al. (2024)
*N. carbonarius*	PDD:113960	New Zealand	PP619110	PP619127	**–**	Douch et al. (2024)
*N. carbonarius*	PDD:118505	New Zealand	PP619121	PP619131	**–**	Douch et al. (2024)
***N. chebalingensis***	**GDGM81341 (HT)**	**China (Guangdong)**	**PZ326307**	**PZ317569**	**PZ339034**	**This study**
*N. colombiensis*	Vasco 2084 (HT)	Colombia	KP972654	**–**	**–**	Grupe et al. (2016)
*N. melanocarpus*	Dai 19964 (HT)	China	**–**	OR814056	**–**	Song et al. (2025)
*N. melanocarpus*	Dai 19978 (ET)	China	OR814013	OR814057	**–**	Song et al. (2025)
***N. melanocarpus***	**GDGM95044**	**China (Hunan)**	**PZ326310**	**PZ317572**	**PZ339036**	**This study**
***N. nanlingensis***	**GDGM80321 (HT)**	**China (Guangdong)**	**PZ326306**	**–**	**PZ339033**	**This study**
*N. pakaraimensis*	Henkel 9554 (ET)	Guyana	KM668103	**–**	**–**	Grupe et al. (2015)
*N. pakaraimensis*	TH9513 (HT)	Guyana	KC155390	**–**	**–**	Grupe et al. (2015)
*N. pallidogriseus*	AV989 (HT)	Colombia	KR698939	**–**	**–**	Grupe et al. (2016)
*N. portoricensis*	Baroni 8776 (HT)	Puerto Rico	KM668100	**–**	**–**	Grupe et al. (2015)
*N. quercophilus*	CFMR-BZ-3833 (HT)	Belize	KM668101	**–**	**–**	Grupe et al. (2015)
*N. rufobrunneus*	Vasco 1989 (HT)	Colombia	KR698937	**–**	**–**	Grupe et al. (2016)
*Neosarcodon* sp.	Yuan19437	China	PV221947	PV257885	**–**	Direct Submission
*N. umbilicatus*	Baroni 10201 (HT)	Belize	KM668102	**–**	**–**	Grupe et al. (2015)
***N. velutinus***	**GDGM98715 (HT)**	**China (Guangdong)**	**PZ326311**	**PZ317573**	**PZ339037**	**This study**
***N. velutinus***	**GDGM98721**	**China (Guangdong)**	**PZ326313**	**PZ317575**	**PZ339039**	**This study**
***N. velutinus***	**GDGM99600**	**China (Guangdong)**	**PZ326314**	**PZ317576**	**PZ339040**	**This study**
*Phellodon atroardesiacus*	Cui16951	China	MZ225632	MZ225597	MZ343197	Song et al. (2021)
*P. cinereofuscus*	Cui16962 (HT)	China	MZ225583	MZ225605	MZ343200	Song et al. (2021)
*P. griseofuscus*	Cui18544 (ET)	China	OL449265	OL439035	OL449087	Song et al. (2022)
*P. melaleucus*	Cui16242	China	OP863058	OP860999	**–**	Song et al. (2022)
*P. perchocolatus*	Cui18536 (HT)	China	OL449260	OL439030	**–**	Song et al. (2022)
*P. stramineus*	Cui16959 (HT)	China	MZ225588	NG_088236	MZ343204	Song et al. (2021)
*P. yunnanensis*	Cui17129 (HT)	China	MZ225594	NG_088237	MZ343207	Song et al. (2021)
*Sarcodon flavidus*	SAAS 3832	China	OK636112	OP407699	OP434381	Wang et al. (2024)
*S. flavidus*	SAAS 3923 (ET)	China	OK636113	OP407700	OP434382	Wang et al. (2024)
*S. giganteus*	SAAS 3576 (HT)	China	OK636121	OP407701	OP434383	Wang et al. (2024)
*S. giganteus*	SAAS 3974 (ET)	China	OK636122	**–**	OP434384	Wang et al. (2024)
*S. imbricatus*	SAAS 2681	China	OK636080	OP407703	OP434385	Wang et al. (2024)
*S. imbricatus*	SAAS 2789	China	OK636082	OP407704	OP434386	Wang et al. (2024)
*S. imbricatus*	SAAS 2859	China	OK636086	OP407705	OP434387	Wang et al. (2024)
*S. leucopus*	SAAS 2873	China	OK636090	OP407706	OP434388	Wang et al. (2024)
*S. leucopus*	SAAS 2875	China	OK636087	OP407707	OP434389	Wang et al. (2024)
*S. nigrosquamosus*	SAAS 2704 (ET)	China	OK636106	OP407709	**–**	Wang et al. (2024)
*S. nigrosquamosus*	SAAS 2938 (ET)	China	OK636101	OP407710	**–**	Wang et al. (2024)
*S. nigrosquamosus*	SAAS 3922 (ET)	China	OK636103	OP407711	**–**	Wang et al. (2024)

## Results

### Molecular phylogenetic results

**Figure 1.**
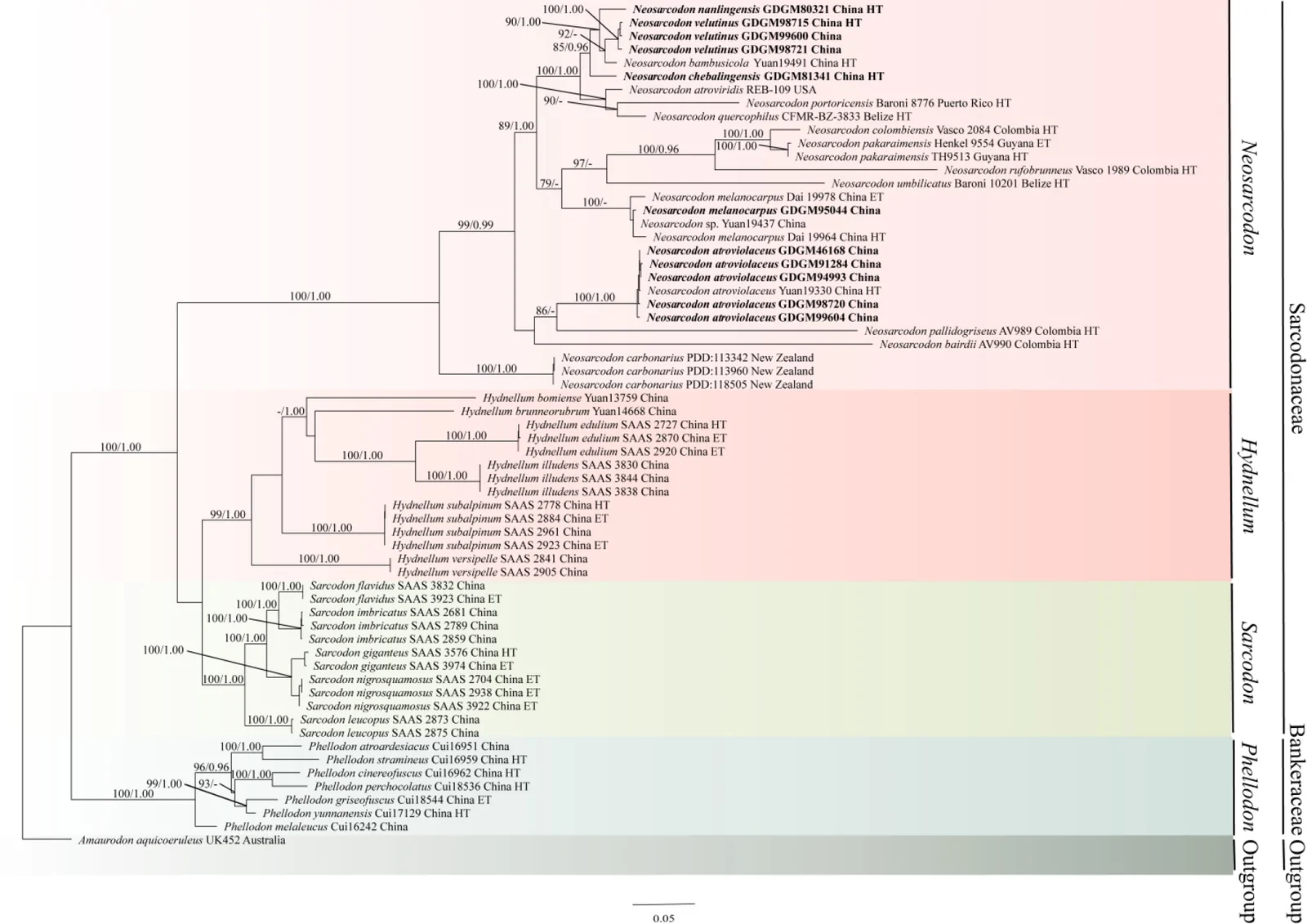
Maximum Likelihood (ML) analysis was performed for the genus *Neosarcodon*, based on concatenated nrITS-nrLSU-*rpb2* sequences. *Amaurodon aquicoeruleus* was selected as the outgroup. Phylogenetic nodes were annotated with MLBS ≥ 75% and BPP ≥ 0.95. Holotypes and epitypes are indicated as HT and ET, respectively. New sequences generated in this study are shown in bold.

A total of 119 sequences were downloaded from the GenBank database for phylogenetic analyses, including 51 nrITS, 41 nrLSU and 27 *rpb2* sequences. Additionally, 30 sequences were newly generated in the present study, comprising 11 nrITS, 10 nrLSU and 9 *rpb2* sequences. The final concatenated nrITS-nrLSU-*rpb2* dataset included 62 nrITS, 50 nrLSU and 36 *rpb2* sequences. The aligned concatenated dataset consisted 2954 nucleotide sites including gaps (875 for nrITS, 1376 for nrLSU and 703 for *rpb2*), with 1837 constant sites, 1333 distinct patterns, 981 parsimony-informative characters and 136 singleton sites. For the concatenated dataset, HKY+F+I+G4, GTR+F+I+G4 and GTR+F+I+G4 were selected as the best-fit substitution models for the BI analysis of nrITS, nrLSU and *rpb2* regions, respectively; whereas TIM2+F+I+G4, GTR+F+I+G4 and TIM2+F+G4 were selected for the ML analysis (Kalyaanamoorthy et al. 2017). The topologies of the phylogenetic trees generated from BI and ML analyses were nearly identical; therefore, only the ML tree is presented (Fig. 1).

In the phylogenetic tree (Fig. 1), *Neosarcodon* is strongly supported as a monophyletic clade (MLBS/BPP = 100%/1.00) and the samples obtained in the present study form five distinct monophyletic lineages within *Neosarcodon*. Amongst these, GDGM80321, GDGM81341, GDGM98715, GDGM99600 and GDGM98721 represent three distinct species lineages closely related to *N. bambusicola*; while, GDGM95044 is nested within the *N. melanocarpus* clade and GDGM46168, GDGM91284, GDGM94993, GDGM98720 and GDGM99604 cluster together with *N. atroviolaceus*.

### Taxonomy

#### Neosarcodon chebalingensis

Taxon classificationFungiThelephoralesBankeraceae

Ming Zhang, X.Y. Yang & C.Q. Wang sp. nov.

D9906CBE-16B4-580B-87A0-8A8F922003B7

Fungal Names: FN 573837

Figs 2A, 3A, 3B, 4

##### Chinese name.

车八岭新肉齿菌 (Che Ba Ling Xin Rou Chi Jun).

**Figure 2.**
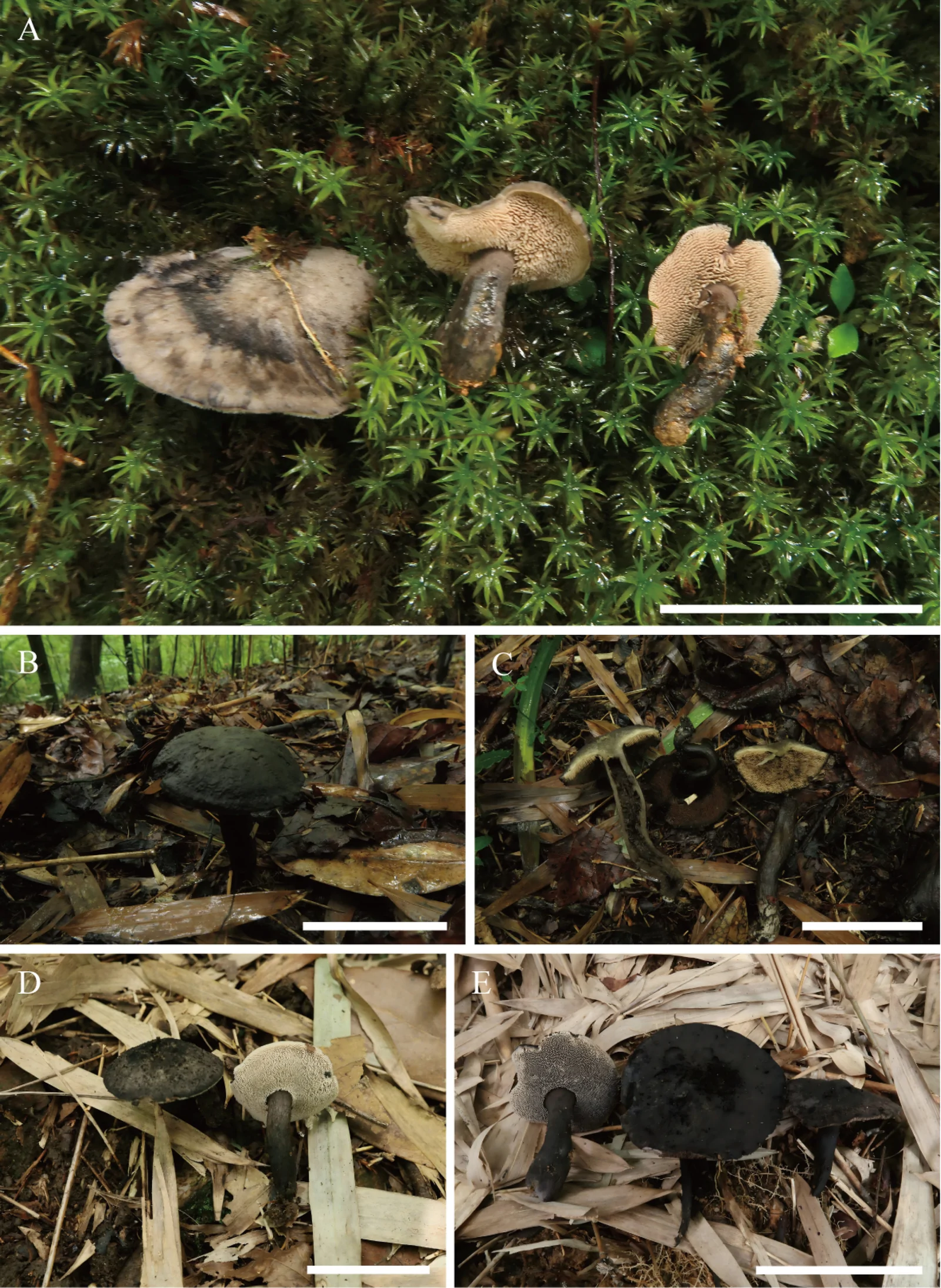
Basidiomata of new *Neosarcodon* species. **A**. *Neosarcodon chebalingensis* (GDGM 81341); **B, C**. *Neosarcodon nanlingensis* (GDGM 80321); **D, E**. *Neosarcodon velutinus* (GDGM 98715). Scale bars: 50 mm (**A–E**).

**Figure 3.**
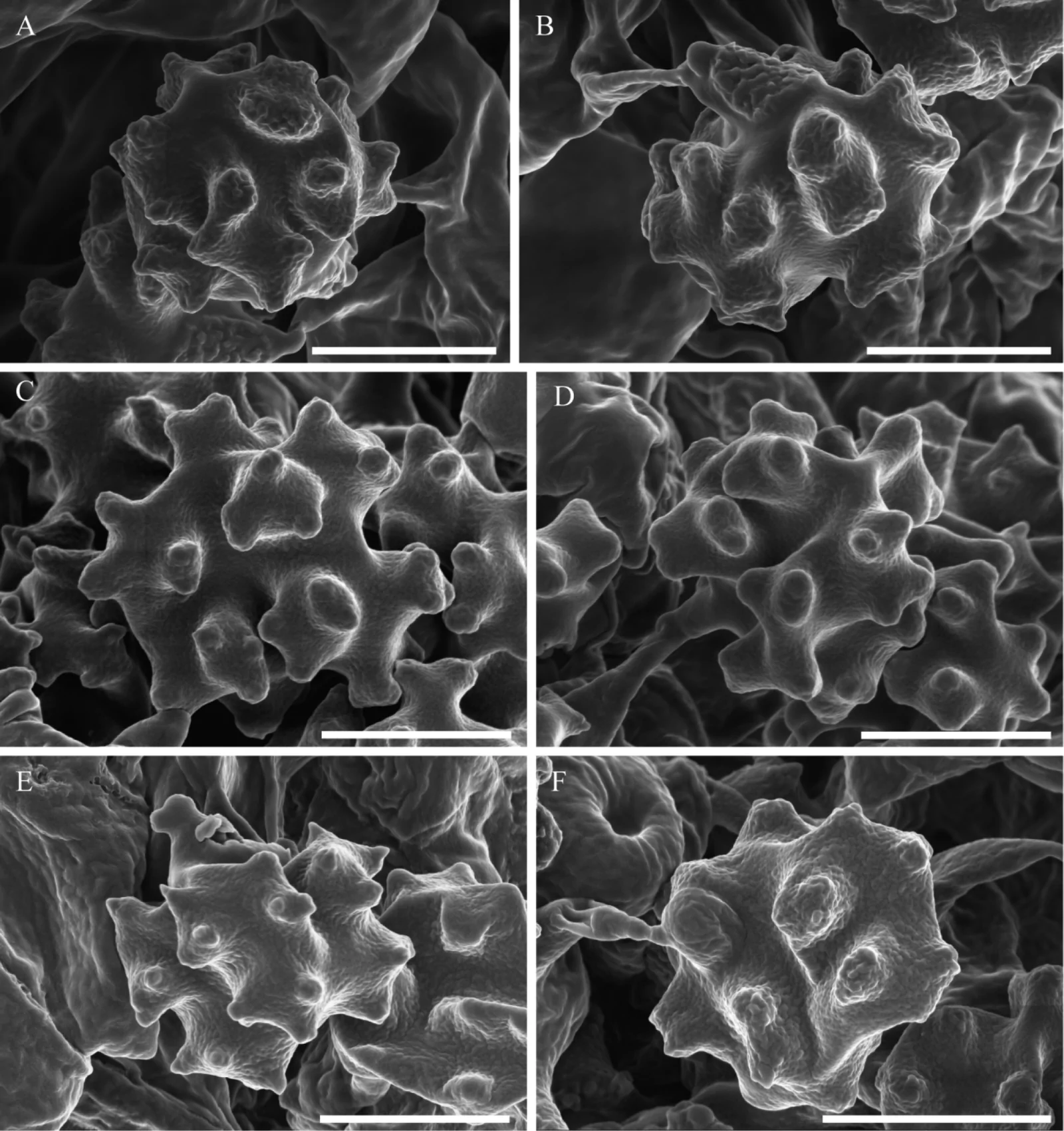
Scanning Electron Microscope (SEM) of basidiospores of new *Neosarcodon* species. **A, B**. *Neosarcodon chebalingensis* (GDGM 81341); **C, D**. *Neosarcodon nanlingensis* (GDGM 80321); **E, F**. *Neosarcodon velutinus* (GDGM 98715). Scale bars: 4 μm (**A–F**).

**Figure 4.**
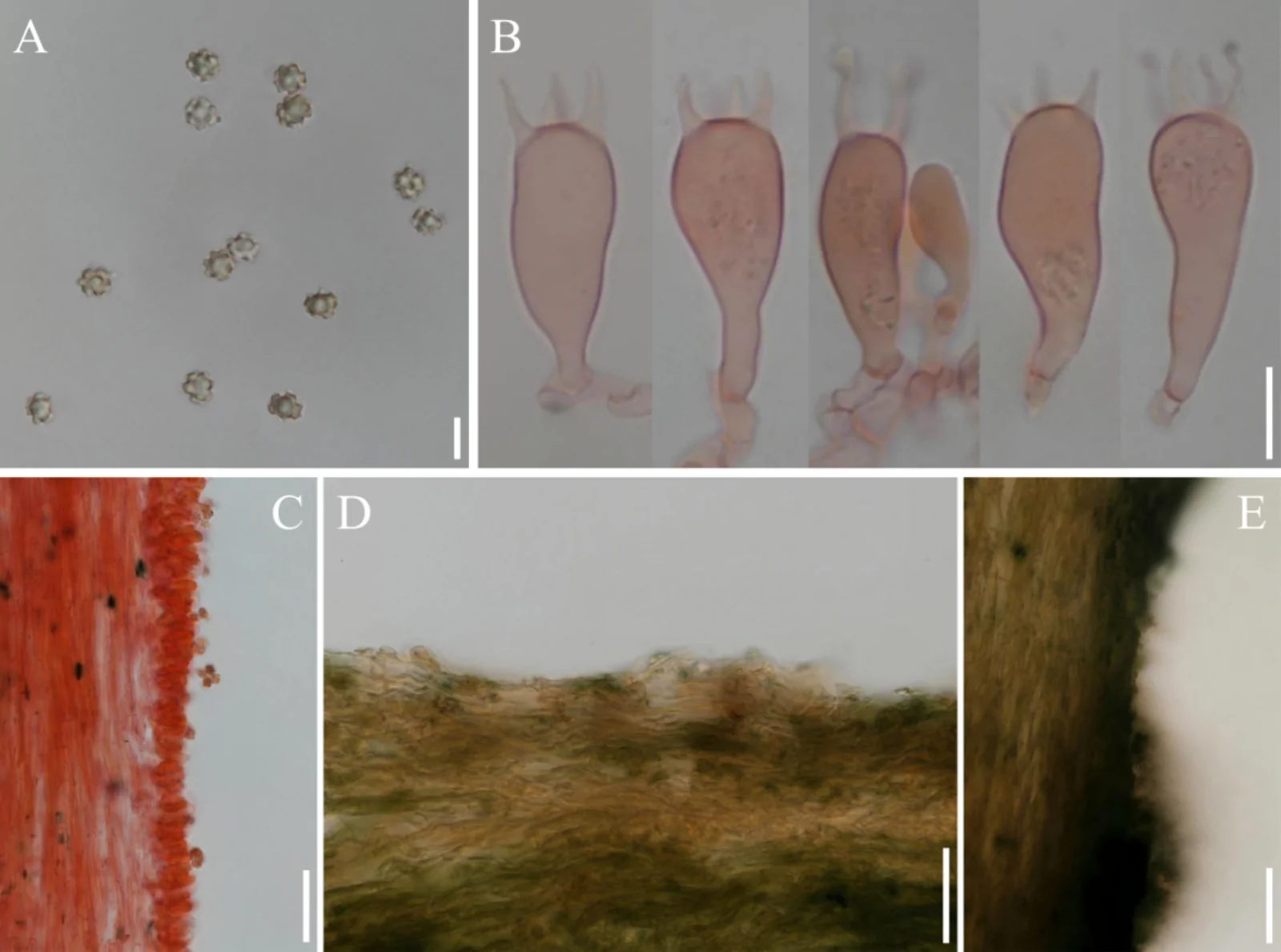
Light microscopy of *Neosarcodon chebalingensis*. (GDGM 81341) **A**. Basidiospores; **B**. Basidia; **C**. Hymenophoral trama; **D**. Pileipellis; **E**. Stipitipellis. Scale bars: 10 μm (**A, B**); 50 μm (**C–E**).

##### Diagnosis.

Similar to *N. atroviolaceus*, but differs by the orange-grey to brownish-grey pileus, larger basidiospores 7.5–10.5 × (7)7.5–9.5(10) μm and different DNA sequences.

##### Holotype.

China • Guangdong Province: Shaoguan City, Guangdong Chebaling National Nature Reserve, at 25.2000°N, 114.4167°E, elev. ca. 380 m, 7 June 2020, Ming Zhang (GDGM 81341, holotype!). GenBank accession numbers: PZ326307 (nrITS); PZ317569 (nrLSU); PZ339034 (*rpb2*).

##### Etymology.

“*chebaling*” refers to the type locality of the new species, located in Chebaling National Nature Reserve, Guangdong, China.

##### Description.

***Pileus*** 20–45 mm broad, plano-convex to applanate, surface dry, glabrous to subtomentose, orange grey to brownish grey (5B2–5C2), usually with cocoa brown to brownish grey tinge (6E5–6F2). ***Spines*** 2–3 × 0.5–1 mm, adnate, soft, conical, dense, orange grey (5B2), changing to bluish black (20D3–20E3) when bruised. ***Context*** 3–6 mm thick, orange grey (5B2), changing to bluish grey (20D3–20E3) when exposed. ***Stipe*** 20–45 × 5–12 mm, subcylindrical, central, solid, glabrous, brown grey to greyish-brown (6E2–4). ***Annulus*** absent. Taste bitter. Odour unpleasant when dried.

***Basidiospores*** 7.5–10.5 × (7)7.5–9.5(10) μm, L_m_ = 8.79 ± 0.82 μm, W_m_ = 8.31 ± 0.66 μm, Q = 1–1.18(1.24), Q_m_ = 1.06 ± 0.05, subglobose, with tuberculate projections, slightly thick-walled, yellowish-brown in KOH, usually contain guttae, inamyloid. ***Basidia*** 27–45 × 9–15.5 μm, clavate to broadly clavate, thin-walled, yellowish-brown in KOH, usually contain guttule, 4-spored, sterigmata 5–7.5 μm long. ***Cystidia*** absent. ***Hymenophoral trama*** regular, composed of cylindrical hyphae 6–13 μm wide, thin-walled, yellowish-brown in KOH, commonly with dark-turquoise crystalline pigments in KOH. ***Pileipellis*** a cutis, composed of cylindrical hyphae 8–15 μm wide, thin-walled, yellowish-brown to olive-green in KOH. ***Stipitipellis*** a cutis, composed of cylindrical hyphae 3–7.5 μm wide, thin-walled, olive-green to black in KOH. ***Clamp connections*** present in all parts.

##### Habit and distribution.

Solitary or scattered on the moss layer under broad-leaved forests dominated by bamboos *Fagaceae* trees in the subtropical region of China. Currently only known from Guangdong Province, China.

##### Notes.

Morphologically, *N. chebalingensis* can be distinguished by its orange-grey to brownish-grey pileus tinged with cocoa-brown to brownish-grey zonate, orange-grey spines changing to bluish-black when bruised, orange-grey context changing to bluish-grey when exposed, grey to soot-brown stipe and subglobose basidiospores, measuring 7.5–10.5 × (7)7.5–9.5(10) μm.

Phylogenetic analyses show that *N. chebalingensis* is closely related to *N. nanlingensis*, *N. velutinus* and *N. bambusicola*. However, *N. nanlingensis* differs by its larger basidiomata (pileus up to 55 mm broad), grey to greyish-brown pileus, dark-blond to oak-brown spines changing to dark-grey when bruised, yellow-grey context changing to dark-grey when exposed. *Neosarcodon bambusicola* differs by its greyish-green to brown pileus, violet-grey spines, dark-violet stipe covered with reticulated fibre, yellow-grey context changing dark-grey when exposed and shorter basidia (18–29 × 6–10 μm) (Zhu et al. 2026). *Neosarcodon velutinus* differs by its grey to greyish-brown pileus with a velvety surface, greenish-grey to greyish-green spines changing to dull-violet to greyish-violet when bruised, dark-grey to greyish-brown stipes covered with reticulated fibres and smaller basidiospores, measuring 6.5–8.5(9) × 5.5–8 μm.

#### Neosarcodon nanlingensis

Taxon classificationFungiThelephoralesBankeraceae

Ming Zhang, X.Y. Yang & C.Q Wang sp. nov.

94A79B38-BE94-5DC1-8D06-50EDDC49A6E3

Fungal Names: FN 573838

**Figure 5.**
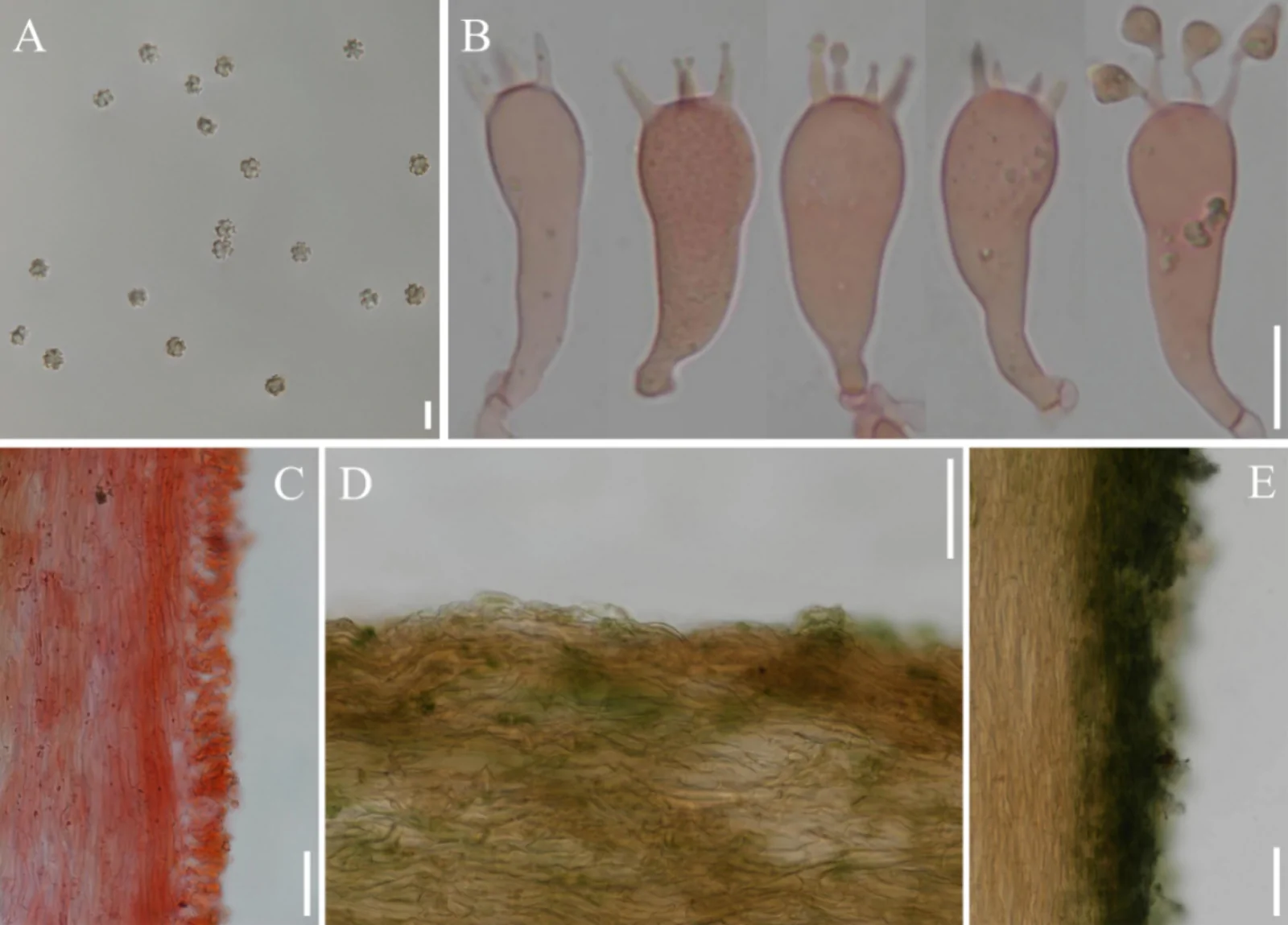
Light microscopy of *Neosarcodon nanlingensis*. (GDGM 80321). **A**. Basidiospores; **B**. Basidia; **C**. Hymenophoral trama; **D**. Pileipellis; **E**. Stipitipellis. Scale bars: 10 μm (**A, B**); 50 μm (**C–E**).

Figs 2B, 2C, 3C, 3D, 5

##### Chinese name.

南岭新肉齿菌 (Nan Ling Xin Rou Chi Jun).

##### Diagnosis.

Similar to *N. bambusicola*, but differs by its grey to greyish-brown pileus, glabrous stipe, larger basidiospores measuring 8–10(11) × (7.5)8–9.5(10) μm and the different DNA sequences.

##### Holotype.

China • Guangdong Province: Shaoguan City, Guangdong Nanling National Nature Reserve, at 25.5833°N, 115.6333°E, elev. ca.1370 m, 9 June 2020, Ming Zhang (GDGM 80321, holotype!). GenBank accession numbers: PZ326306 (nrITS); PZ339033 (*rpb2*).

##### Etymology.

*“Nanling”* refers to the type locality of the new species, located in Nanling National Nature Reserve, Guangdong, China.

##### Description.

***Pileus*** 40–55 mm broad, plano-convex to convex, surface dry, glabrous, grey to greyish brown (5F1–3). ***Spines*** 2–3 × 0.5–1 mm, adnate, soft, conical, dense, dark blond to oak brown (5D4–6), changing to dark grey (1F6–8) when bruised. ***Context*** 5–8 mm thick, ash grey to greyish yellow (2B2–3), changing to dark grey (1F6–8) when exposed. ***Stipe*** 50–70 × 5–13 mm, central, broadly fistulose, subcylindrical, glabrous, grey to soot brown (5F1–5). ***Annulus*** absent. Taste bitter. Odour unpleasant when dried.

***Basidiospores*** 8–10(11) × (7.5)8–9.5(10) μm, L_m_ = 9 ± 0.60 μm, W_m_ = 8.59 ± 0.54 μm, Q = 1–1.13, Q_m_ = 1.05 ± 0.04, subglobose, with tuberculate projections, slightly thick-walled, yellowish-brown in KOH, usually contain guttule, inamyloid. ***Basidia*** 20–42 × 8.5–13 μm, clavate to broadly clavate, thin-walled, yellowish-brown in KOH, usually contain guttae, 4-spored, sterigmata 5–7.5 μm long. ***Cystidia*** absent. ***Hymenophoral trama*** regular, composed of cylindrical hyphae 6.5–14 μm wide, thin-walled, yellowish-brown in KOH, commonly with dark-turquoise crystalline pigments in KOH. ***Pileipellis*** a cutis, composed of cylindrical hyphae 6.5–13.5 μm wide, thin-walled, yellowish-brown to olive-green in KOH. ***Stipitipellis*** a cutis, composed of cylindrical hyphae 5–17 μm wide, thin-walled, yellowish-brown to black in KOH. ***Clamp connections*** present in all parts.

##### Habit and distribution.

Solitary or scattered under mixed forests dominated by bamboos, with fewer *Fagaceae* trees in subtropical regions of southern China. Currently only known from Guangdong Province, China.

##### Notes.

Morphologically, *N. nanlingensis* can be distinguished by its grey to greyish-brown pileus, dark-blond to oak-brown spines, grey to brownish-grey context changing to dark-grey when exposed, grey to soot-brown stipe, subglobose basidiospores measuring 8–10(11) × (7.5)8–9.5(10) μm and distributed in subtropical broad-leaf forests dominated by bamboo. *Neosarcodon melanocarpus*, recently reported from southwest China, is similar to *N. nanlingensis*; however, *N. melanocarpus* differs in having smaller basidiomata (pileus up to 2 cm broad), black pileus, brown to greyish-brown spines and thick-walled hyphal system (Song et al. 2025). *Neosarcodon pakaraimensis* is also similar to *N. nanlingensis*, but differs in having larger basidiomata (pileus up to 72 mm broad), grey to greyish-brown pileus changing to black when bruised, cream to dark-brown spines and smaller basidiospores measuring 5–7(8) × 5–9(10) µm (Grupe et al. 2015); Additionally, *N. pakaraimensis* is currently only known from the Neotropics Guyana, under *Pakaraimaea*/*Dicymbe* forests (Grupe et al. 2015), while *N. nanlingensis* distributed in subtropical regions of southern China under bamboo forests.

Phylogenetic analyses shown that *N. nanlingensis* is closely related to *N. bambusicola* and *N. velutinus*. However, *N. bambusicola* differs by its greyish-green to brown basidiomata, violet-grey spines, dark-violet stipe and smaller basidia measuring 18–29 × 6–10 μm (Zhu et al. 2026). *Neosarcodon velutinus* differs by its velvety pileus, greenish-grey to greyish-green spines, dark-grey to greyish-brown stipe covered with reticulated fibres, grey to brownish-grey context changing to dull-violet to greyish-violet when exposed and smaller basidiospores measuring 6.5–8.5(9) × 5.5–8 μm.

#### Neosarcodon velutinus

Taxon classificationFungiThelephoralesBankeraceae

Ming Zhang, X.Y. Yang & C.Q. Wang sp. nov.

0F854632-38F1-51C9-AAC0-11C5A266B527

Fungal Names: FN 573839

**Figure 6.**
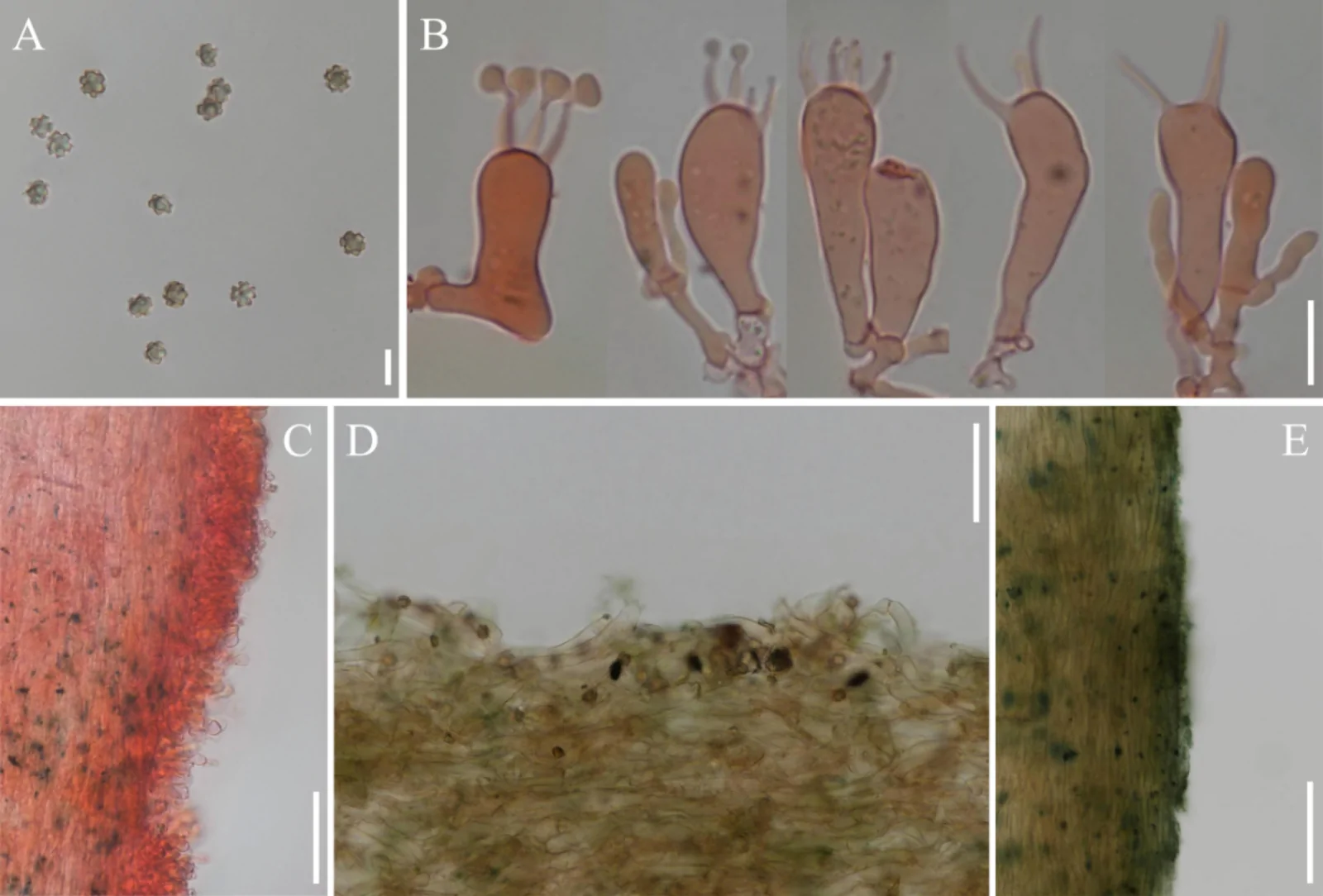
Light microscopy of *Neosarcodon velutinus* (GDGM 98715). **A**. Basidiospores; **B**. Basidia; **C**. Hymenophoral trama; **D**. Pileipellis; **E**. Stipitipellis. Scale bars: 10 μm (**A, B**); 50 μm (**C–E**).

Figs 2D, 2E, 3E, 3F, 6

##### Chinese name.

绒盖新肉齿菌 (Rong Gai Xin Rou Chi Jun).

##### Diagnosis.

Similar to *N. melanocarpus*, but differs by its relatively larger basidiomata, violet-grey to dark-violet, velvety pileus, grey to brownish-grey context changing to dull-violet to greyish-violet when exposed and thin-walled hyphae of pileipellis.

##### Holotype.

China • Guangdong Province: Shaoguan City, Guangdong Chebaling National Nature Reserve, elev. ca. 700 m, at 24.7142°N, 114.2050°E, 7 June 2025, Ming Zhang, Xinyue Yang and Liuying An (GDGM 98715, holotype!). GenBank accession numbers: PZ326311 (nrITS); PZ317573 (nrLSU); PZ339037 (*rpb2*).

##### Etymology.

The Latin epithet *“velutinus”* = velutinous, refers to the velvety pileus surface of the species.

##### Description.

***Pileus*** 25–55 mm broad, hemispherical when young, plano-convex to convex when mature; surface dry, velutinous, violet grey to dark violet (15F2–4). ***Spines*** 2–3 × 0.5–1 mm, adnate, soft, conical, dense, golden grey to brownish grey (4C2–5D2), changing to dull violet to greyish violet (18E3–6) when bruised. ***Context*** 5–10 mm thick, grey to brownish grey (5D1–2), changing to dull violet to greyish violet (18E3–6) when exposed. ***Stipe*** 25–60 × 5–10 mm, subcylindrical, base swollen, central, solid, brownish grey to fawn brown (7F2–4). ***Annulus*** absent. Taste bitter. Odour disagreeable when dried.

***Basidiospores*** 6.5–8.5(9) × 5.5–8 μm, L_m_ = 7.58 ± 0.57 μm, W_m_ = 6.91 ± 0.63 μm, Q = 1–1.27(1.33), Q_m_ = 1.10 ± 0.08, subglobose to broadly ellipsoid, with tuberculate or spinose projections, slightly thick-walled, yellowish-brown in KOH, usually contain guttae, inamyloid. ***Basidia*** 20–47.5 × 8.5–16 μm, clavate to broadly clavate, thin-walled, yellowish-brown in KOH, usually contain guttae, 4-spored, occasionally 2-spored, sterigmata 4.5–12.5 μm long. ***Hymenophoral trama*** regular, composed of cylindrical hyphae 4.5–21 μm wide, thin-walled, light-brown in KOH, commonly with dark-turquoise crystalline pigments in KOH. ***Pileipellis*** a cutis, partly with up turned terminal hyphae, composed of cylindrical hyphae 5–9 μm wide, thin-walled, yellowish-brown to olive-green in KOH. ***Stipitipellis*** a cutis, composed of cylindrical hyphae 5–9 μm wide, thin-walled, yellowish-brown to olive green in KOH. ***Clamp connections*** present in all parts.

##### Habit and distribution.

Solitary or scattered under mixed forests dominated by bamboos, with fewer *Fagaceae* trees in subtropical regions of southern China. Currently only known from Guangdong Province, China.

##### Additional specimen examined.

China • Guangdong Province: Shaoguan City, Chebaling National Nature Reserve, at 24.7124°N, 114.2036°E, elev. ca. 680 m, 7 June 2025, Ming Zhang, Xinyue Yang and Liuying An (GDGM98721), GenBank accession numbers: PZ326313 (nrITS); PZ317575 (nrLSU); PZ339039 (*rpb2*). Same location, at 24.7097°N, 114.1614°E, elev. ca. 530 m, 8 June 2025, Guorui Zhong and Ruihua Sun (GDGM99600), GenBank accession numbers: PZ326314 (nrITS); PZ317576 (nrLSU); PZ339040 (*rpb2*).

##### Notes.

Morphologically, *N. velutinus* can be distinguished by its violet-grey to dark-violet pileus, brownish-grey to fawn-brown stipe, golden-grey to brownish-grey spines, grey to brownish-grey context changing to dull-violet to greyish-violet when exposed, subglobose to broadly ellipsoid basidiospores and thin-walled terminal hyphae of pileipellis. *Neosarcodon bambusicola* and *N. melanocarpus*, recently reported from southwest China, resemble *N. velutinus* in morphology. However, *N. bambusicola* differs in having greyish-green to brown pileus, violet-grey spines, dark-violet stipe and smaller basidia (18–29 × 6–10 μm) (Zhu et al. 2026); *N. melanocarpus* differs in having smaller basidiomata (pileus up to 2 cm broad), glabrous pileus and larger basidiospores (6.5–9 × 6–8.5 µm, without ornamentation) (Song et al. 2025).

## Discussion

Notably, *Neosarcodon* has long been regarded as a predominantly Neotropical genus, while its occurrence in the Old World, particularly in East Asia, remained poorly documented (Grupe et al. 2015, 2016; Douch et al. 2024). Before the taxonomic establishment and circumscription of *Neosarcodon*, no species of the genus had been recorded from China (Wang et al. 2024). This knowledge gap may be partly attributed to the limited number of known species, the subtle diagnostic morphological characteristics, difficulties in species identification, insufficient field sampling and a lack of molecular phylogenetic studies in the relevant regions.

Together with the three previously known Chinese species, the three new species described in this study are also distributed in subtropical southern China. This finding indicates that the occurrence of *Neosarcodon* in southern China may have been substantially underestimated. When integrated with previous records, these results indicate that subtropical regions of southern China may represent an important species diversity centre of *Neosarcodon*. However, this hypothesis requires further validation through more extensive geographic sampling and more comprehensive phylogenetic analyses.

The species reported from Asia, especially from China, not only enrich the known species diversity of *Neosarcodon*, but also provide new insights into its evolutionary and biogeographic history. Phylogenetic analyses indicate that these Asian species are nested within clades composed of Neotropical *Neosarcodon* species, implying that the genus may have a broader historical distribution or a more complex biogeographic history than previously assumed. Such a pattern may reflect historical dispersal, shared ancestry, ancient divergence and/or long-distance dispersal events. Although these hypotheses require further evaluation through expanded taxon sampling, molecular dating and formal biogeographic analyses, the present phylogenetic evidence provides valuable clues for reconstructing the historical biogeography of the genus.

Currently, most known *Neosarcodon* species are ectomycorrhizal fungi that form symbiotic relationships with various plants. Interestingly, two new species discovered in this study, i.e. *N. nanlingensis* and *N. velutinus* and recently published species *N. atroviolaceus* and *N. bambusicola* were found in bamboo forests (Zhu et al. 2026). This leads us to ponder whether there is a symbiotic relationship between *Neosarcodon* and bamboos and whether the life style of this genus has undergone a transformation from forming ectomycorrhizal symbiosis with broad-leaved trees to forming symbiosis with gramineous bamboos. Thus, the trophic status of the these *Neosarcodon* species warrant further investigation.

### A Key to Chinese species of *Neosarcodon*

**Table:** 

1	Basidiomata small-sized; pileus ≤ 2 cm broad, black; basidiospores 6.5–9 × 6–8.5 µm (without the ornamentation)	***Neosarcodon melanocarpus***
–	Basidiomata medium-sized to large-sized; pileus usually > 2 cm broad	**2**
2	Pileus orange-grey to brownish-grey, glabrous to subtomentose; basidiospores 7.5–10.5 × (7)7.5–9.5(10) μm	***Neosarcodon chebalingensis***
–	Pileus without orange tinge	**3**
3	Basidospores up to 10 μm long; pileus grey to greyish-brown, glabrous; basidiospores 8–10(11) × (7.5)8–9.5(10) μm	***Neosarcodon nanlingensis***
–	Basidospores usually less than 10 μm long	**4**
4	Pileus not glabrous, violet-grey to dark-violet, velutinous; basidiospores 6.5–8.5(9) × 5.5–8 μm	***Neosarcodon velutinus***
–	Pileus glabrous; stipe with reticulated fibre	**5**
5	Basidiomata dark-violet; hymenophoral trama thin- to slightly thick-walled; basidiospores 5–6 × (4.5–)5–5.5(–6) μm	***Neosarcodon atroviolaceus***
–	Basidiomata greyish-green to brown; hymenophoral trama thin-walled; basidiospores (4)4.5–5 × 3–4.5 μm	***Neosarcodon bambusicola***

## Supplementary Material

XML Treatment for Neosarcodon chebalingensis

XML Treatment for Neosarcodon nanlingensis

XML Treatment for Neosarcodon velutinus

## Additional information

### Artificial Intelligence (AI) use

The authors accept full responsibility for the content of the manuscript, including the disclosure of any use of AI.

No AI tools were used in the preparation of this manuscript.

### Author ORCIDs

X.-Y. Yang https://orcid.org/0009-0001-2072-3615

L.-Q. Deng https://orcid.org/0009-0007-7666-9607

Y.-M. Zhang https://orcid.org/0009-0002-7138-7647

W.-Q. Deng https://orcid.org/0000-0001-8790-2549

H.-M. Yue https://orcid.org/0000-0001-9282-2295

C.-Q. Wang https://orcid.org/0000-0001-8665-0618

M. Zhang https://orcid.org/0000-0001-6420-2531

### Data availability

All of the data that support the findings of this study are available in the main text.

## References

[B1] Douch JK, Vaughan LJ, Cooper JA, Holmes GD, Robinson R, Stefani F, Idnurm A, May TW (2024) Taxonomic revision of fleshy species of *Hydnellum*, *Neosarcodon*, and *Sarcodon* (*Thelephorales*) from Australasia. Mycologia 116: 965–992. 10.1080/00275514.2024.2363211 39208239

[B2] Grupe AC, Baker AD, Uehling JK, Smith ME, Baroni TJ, Lodge DJ, Henkel TW (2015) *Sarcodon* in the Neotropics I: new species from Guyana, Puerto Rico and Belize. Mycologia 107: 591–606. 10.3852/14-185 25661714

[B3] Grupe AC, Vasco-Palacios AM, Smith ME, Boekhout T, Henkel TW (2016) *Sarcodon* in the Neotropics II: four new species from Colombia and a key to the regional species. Mycologia 108: 791–805. 10.3852/15-254 27091388

[B4] Kalyaanamoorthy S, Minh BQ, Wong TKF, von Haeseler A, Jermiin LS (2017) ModelFinder: fast model selection for accurate phylogenetic estimates. Nature Methods 14(6): 587–589. 10.1038/nmeth.4285 PMC5453245 28481363

[B5] Katoh K, Standley DM (2013) MAFFT multiple sequence alignment software version 7: improvements in performance and usability. Molecular Biology and Evolution 30(4): 772–780. 10.1093/molbev/mst010 PMC3603318 23329690

[B6] Kornerup A, Wanscher JH (1978) Methuen Handbook of Colour. 3^rd^ edn. Eyre Methuen, London, 252 pp.

[B7] Larsson KH, Svantesson S, Miscevic D, Koljalg U, Larsson E (2019) Reassessment of the generic limits for *Hydnellum* and *Sarcodon* (*Thelephorales*, *Basidiomycota*). Mycokeys 54: 31–47. 10.3897/mycokeys.54.35386 PMC6579789 31231164

[B8] Miettinen O, Kõljalg U (2007) *Amaurodon sumatranus* (*Thelephorales*, *Basidiomycota*), a new species from Indonesia. Mycotaxon 100: 51–59. 10.5962/p.419224

[B9] Minh BQ, Nguyen MA, von Haeseler A (2013) Ultrafast approximation for phylogenetic bootstrap. Molecular Biology and Evolution 30: 1188–1195. 10.1093/molbev/mst024 PMC3670741 23418397

[B10] Mu YH, Yu JR, Cao T, Wang XH, Yuan HS (2021) Multi-Gene Phylogeny and Taxonomy of *Hydnellum* (*Bankeraceae*, *Basidiomycota*) from China. Journal of Fungi 7(10): 818. 10.3390/jof7100818 PMC8540476 34682238

[B11] Nguyen LT, Schmidt HA, von Haeseler A, Minh BQ (2015) IQ-TREE: a fast and effective stochastic algorithm for estimating maximum-likelihood phylogenies. Molecular Biology and Evolution 32(1): 268–274. 10.1093/molbev/msu300 PMC4271533 25371430

[B12] Rambaut A (2018) FigTree. http://tree.bio.ed.ac.uk/software/figtree

[B13] Ronquist F, Teslenko M, Mark P, Ayres DL, Darling A, Höhna S, Larget B, Liu L, Suchard MA, Huelsenbeck JP (2012) MrBayes 3.2: efficient Bayesian phylogenetic inference and model choice across a large model space. Systematic Biology 61(3): 539–542. 10.1093/sysbio/sys029 PMC3329765 22357727

[B14] Song CG, Ji X, Liu S, He XL, Cui BK (2021) Taxonomy and Molecular Phylogeny of *Phellodon (Thelephorales)* with Descriptions of Four New Species from Southwest China. Forests 12: 932. 10.3390/f12070932

[B15] Song CG, Chen YY, Liu S, Xu TM, He XL, Wang D, Cui BK (2022) A phylogenetic and taxonomic study on *Phellodon* (*Bankeraceae*, *Thelephorales*) from China. Journal of Fungi 8: 429. 10.3390/jof8050429 PMC9147675 35628685

[B16] Song CG, Xu TM, Xu YH, Wang D, Zeng L, Fan XP, Sun YF, Cui BK (2025) Systematic revision, molecular phylogeny and divergence times of *Thelephorales (Basidiomycota)*. Mycosphere 16(1): 296–422. 10.5943/mycosphere/16/1/5

[B17] Tamura K, Stecher G, Peterson D, Filipski A, Kumar S (2013) MEGA6: Molecular Evolutionary Genetics Analysis version 6.0. Molecular Biology and Evolution 30: 2725–2729. 10.1093/molbev/mst197 PMC3840312 24132122

[B18] Vilgalys R, Hester M (1990) Rapid genetic identification and mapping enzymatically amplified ribosomal DNA from several *Cryptococcus* species. Journal of Bacteriology 172: 4238–4246. 10.1128/jb.172.8.4238-4246.1990 PMC213247 2376561

[B19] Wang D, Feng H, Zhou J, Liu TH, Zhang ZY, Xu YY, Tang J, Peng WH, He XL (2024) New insights into the stipitate hydnoid fungi *Sarcodon*, *Hydnellum*, and the formerly informally defined *Neosarcodon*, with emphasis on the edible species marketed in Southwest China. IMA Fungus 15: 8. 10.1186/s43008-023-00138-1 PMC10964585 38528625

[B20] White TJ, Bruns TD, Lee SB, Taylor JW, Innis MA, Gelfand DH, Sninsky J (1990) Amplification and Direct Sequencing of Fungal Ribosomal RNA Genes for Phylogenetics. Academic Press, San Diego, 315–322. 10.1016/B978-0-12-372180-8.50042-1

[B21] Zhang M, Huang YX, Xia WX, Li TH, Zhou P, Wang CQ, Deng WQ (2025) Species diversity, taxonomy, distribution and multi-gene phylogeny of chanterelles in China. Mycosphere 16(1): 3753–3950. 10.5943/mycosphere/16/1/30

[B22] Zhao D, Ye T, Gao FL, Jakovlić I, La Q, Tong YD, Liu X, Song R, Liu F, Lian ZM, Zou H, Li WX, Wang GT, Zeng B, Zhang D (2025) PhyloSuite v2: The Development of an All‐in‐One, Efficient and Visualization‐Oriented Suite for Molecular Dating Analysis and Other Advanced Features. IMeta: e70095. 10.1002/imt2.70095 PMC12747537 41472854

[B23] Zhu YQ, Zhou LJ, Liu JX, Yuan YW, Wei YL, Yuan HS (2026) Thirty new species in *Thelephorales* and *Hymenochaetales* from the Eastern Himalayas and Southern China. Mycosphere 17: e001. 10.48130/mycosphere-0026-0001

